# Exploring on the climate regionalization of Qinling-Daba mountains based on Geodetector-SVM model

**DOI:** 10.1371/journal.pone.0241047

**Published:** 2020-11-02

**Authors:** Yufan Hu, Yonghui Yao, Zhixiang Kou

**Affiliations:** 1 State Key Laboratory of Resources and Environmental Information System, Institute of Geographic Sciences and Natural Resources Research, CAS, Beijing, China; 2 University of Chinese Academy of Sciences, Beijing, China; Universiti Teknologi Malaysia, MALAYSIA

## Abstract

Qinling-Daba Mountains (QDM), which are located in central China, are considered as a significant climatic boundary delimiting north and south. However, the influence of complex topographic and climatic features makes it challenging to identify the exact location of the boundary, and different scholars delimit the boundary with significant differences. In addition, there is a gradual transition between climate zones, and no real dividing line exists. To explore the climate regionalization of the QDM, we focused on the identification of the transition zone rather than the exact location of the boundary between subtropical and temperate zones. Thus, we proposed a new workflow for climate regionalization based on the Geodetector-SVM model (a combination of Geodetector and support vector machines). First, we selected the spatial distribution data of six vegetation types (including typical subtropical and temperate vegetation) to represent the spatial distribution of climatic zones. Environmental factors (such as topography, temperature, precipitation, and soil) were used as explanatory variables for the spatial distribution of vegetation. Second, using the Geodetector-SVM model, the distribution characteristics and suitable environment of typical vegetation in different climatic zones are comprehensively explored. By analyzing the multiple boundaries between subtropical and temperate vegetation, the location of the transition zone of the QDM was identified. The results revealed the following: (1) The new workflow for climate regionalization based on the Geodetector-SVM model is powerful for the identification of the transition zone. The q-statistics are generally greater than 0.35, indicating that the transition zone between subtropical and temperate zones can highly reflect the character of the QDM; (2) From west to east, the transition zone mainly passes through the cities of Heishui County, Kang County, Liuba County, and Yichuan County and is approximately 30 km wide.

## Introduction

The process of climate regionalization divides an area into smaller regions that are homogeneous with respect to a specified climatic characteristic. The Köppen-Geiger climate classification [1, 2], which broadly classifies regions by monthly air temperature and precipitation, is one of the most widely used climatic classification systems in the world. It is a global and relatively coarse climate classification scheme. However, the climatic regionalization of Qinling-Daba Mountains (QDM) is extremely challenging due to complex topographical conditions and large mountains. According to the Koppen classification system [3], eight basic climate types are mixed in this region. In fact, QDM has served as the natural geographical boundary of north/south China and the climate boundary of subtropical/temperate zones from a very early time [4, 5]. The accurate division of climatic boundaries was significant in both general and applied climatology [6]. By comprehensive analyzing each element of the environment, the spatial stratified heterogeneity of natural surface in QDM be correctly reflected.

In China, this domain has been explored for more 80 years, resulting in the accumulation of numerous results [7–10]; hundreds of scholars have proposed and developed many subtropical/temperate zone boundary division methods based on climate, vegetation, and soil [3, 11–14]. The location of the subtropical/temperate zone boundary represents an essential issue of Chinese physical geography [10]. Due to the differences in the delimitation indicators and research purposes, the specific location of this climate boundary remains controversial [15]. Generally, there are three main opinions on the location of the climate boundary in QDM: the north slope of Qinling Mountains, the main ridge of Qinling Mountains (the watershed of the Yellow River and the Yangtze River), and the southern slope of the Qinling Mountains at an altitude of 800-1000 m [16]. Zhu Kezhen (1958) proposed that the Qinling-Huaihe river area was the subtropical/temperate boundary [17]. He considered temperature as the key indicator along with temperature-related indicators, including the accumulated temperature, the coldest month average air temperature, etc. Chen Xianji (1982) also delimited the subtropical/temperate boundary in the Qinling-Huaihe river, based on temperature-related indicators. However, Qiu Baojian, Zheng Du, and others suggested that the boundary of the subtropical/temperate zone should be calibrated on the main ridge [18, 19]. In addition to temperature-related indicators, vegetation and soil also serve as auxiliary indexes. However, others hold the opinion that the boundary was mainly located on the southern slope of the Qinling Mountains. Zhang Jinquan suggested that vegetation and soil should be mainly considered because these parameters could better reflect the long-term development of nature. The boundary was mainly located on the southern slope of the Qinling Mountains at an altitude of 800 m [10].

Although much of the controversy is caused by differences in the selection of index systems and the research method of climatic regionalization schemes [20], the most important reason is the complexity, transition, and heterogeneity of QDM. In fact, the process of change from the subtropical zone to temperate zone is gradual, and the boundary of the subtropical/temperate zone cannot be drawn as a line [21, 22]. Scholars were often misled by the term “boundary line” and ignored the properties of the transition zone [23]. The concept of the subtropical/temperate zone boundary should be understood as a wide or narrow transition zone [24]. This is also the reason why different scholars delimited the boundary with significant differences. Therefore, to study and analyze the characteristics of the QDM, focusing on the transition zone rather than the exact location of the boundary between subtropical and temperate zones would be more scientific. To identify the location of the transition zone of the QDM, a new workflow for comprehensive physical regionalization was proposed in this paper.

Methodologically, with the development of machine learning, an increasing number of statistical models used in many studies to define climate regions in the world. These models were mostly used to group databases by the degree of similarity in variability based on temperature- and precipitation-related indicators. Cluster analysis has been employed to climatic regionalization in CONUS [25] and the Turkey [26]. Principal component analysis (PCA) was used by [11, 27] for the construction of homogeneous regions, whereas Sadri and Burn (2011) used Fuzzy C-Means (FCMs) along with L-moments [28]. Each technique has its own advantages and shortcomings [14]. All of these methods have the ability to recognize the subtropical/temperate boundary. However, none of these methods can objectively identify the transition zone. Moreover, climatic regionalization typically corresponds to vegetation distribution in the sense that each climate type is dominated by a vegetation zone or ecological region [29–32]. None of the above methods takes into account the impact of vegetation.

It is possible to use the conjugate relationship between vegetation and climate to indicate the regional zone attributes [33]. Moreover, the sensitivity of various vegetation types to climate differs, and the boundary of typical subtropical/temperate vegetation will be slightly different. For example, the typical subtropical/temperate vegetation boundary of coniferous forest and broad-leaved forest in same area will be different. These differences are the manifestations of the gradual transition characteristics of the transition zone. It is reasonable to delimit the transition based on the boundaries of zonal vegetation of different types (coniferous forest and, broad-leaved forest). Therefore, to identify the location of the transition zone of the QDM, this paper focuses on the delimitation of the boundaries between typical zonal vegetation (typical subtropical vegetation and typical temperate vegetation).

To delimit the transitional zone of subtropical/temperate boundaries, a combination of the Geodetector model and the support vector machine (SVM) was used to identify multiple boundaries between typical zonal vegetations. First, the Geodetector model is a novel tool to measure the degree of spatial stratified heterogeneity [34, 35]. Similarly, the basis of comprehensive physical regionalization is spatial stratified heterogeneity. Xu (2018) used this model to evaluate the indicators of China’s subtropical/tropical zone [36]. The indicator system of covariates for different vegetation types can be built using this model. Second, the goal of our workflow is to identify the boundary between two zonal vegetation type. The SVM model is ideal classifier to solve this problem. Because the SVM model was initially designed for binary classification [37], it can effectively distinguish between two zonal vegetation types. By analyzing the spatial characteristics of numerous boundaries of zonal vegetation, the composition of boundaries can be seen as the transitional zone. Such research can provide a new perspective on the comprehensive physical regionalization of China and reveal the ecological significance of the QDM for China.

## Materials and methods

### Study area

**Fig 1:**
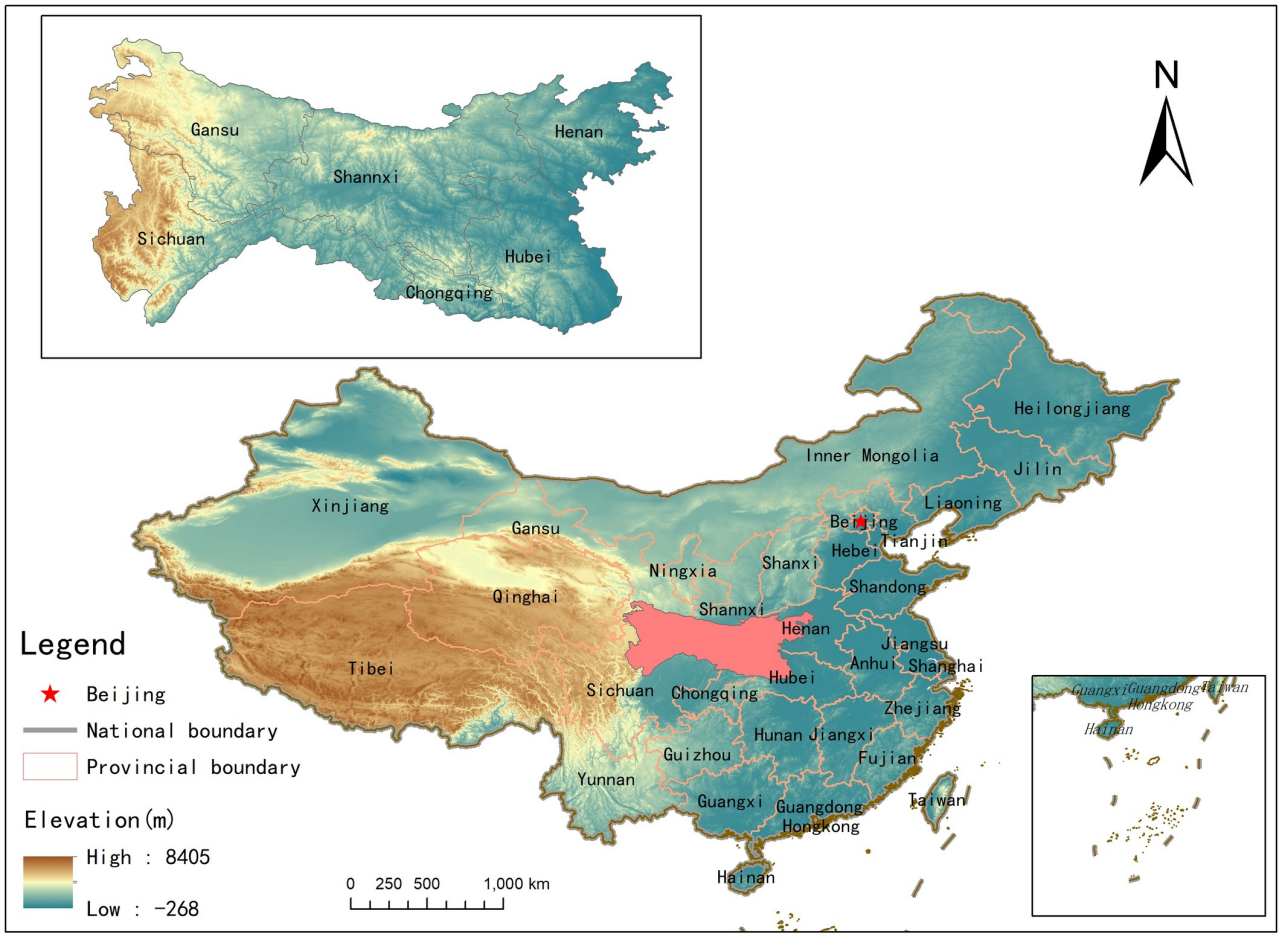
Study area.

QDM, which is situated in the central part of China (30°-36°N, 101°-114°E), is the transitional zone of the subtropical zone and temperate zone (shown in Fig 1). It stretches across six provinces, including Shaanxi, Gansu, Szechwan, Chongqing, Hubei, and Henan, with a land area of 300,000 km² and greater than 1000 km long [38]. Influenced by both the monsoon climate and the continental monsoon climate, its weather is warm and humid, and its environment has a high level of biological diversity [39, 40]. Moreover, characterized by high mountains alternating with deep gorges, the Qinling Mountains serve as the watershed of the Yangtze River and the Yellow River and are regarded as the natural boundary of geography and climate between northern and southern China. Given the integrated influence of horizontal zonality, vertical zonality, aspect and slope, soil environment and even human activities, the environment of QDM is highly complex, diversity and heterogeneous. The spatial distribution of vegetation in the QDM exhibits high spatial stratified heterogeneity with distinct transitional characteristics.

### Data source

#### Spatial distribution data of vegetation

**Fig 2:**
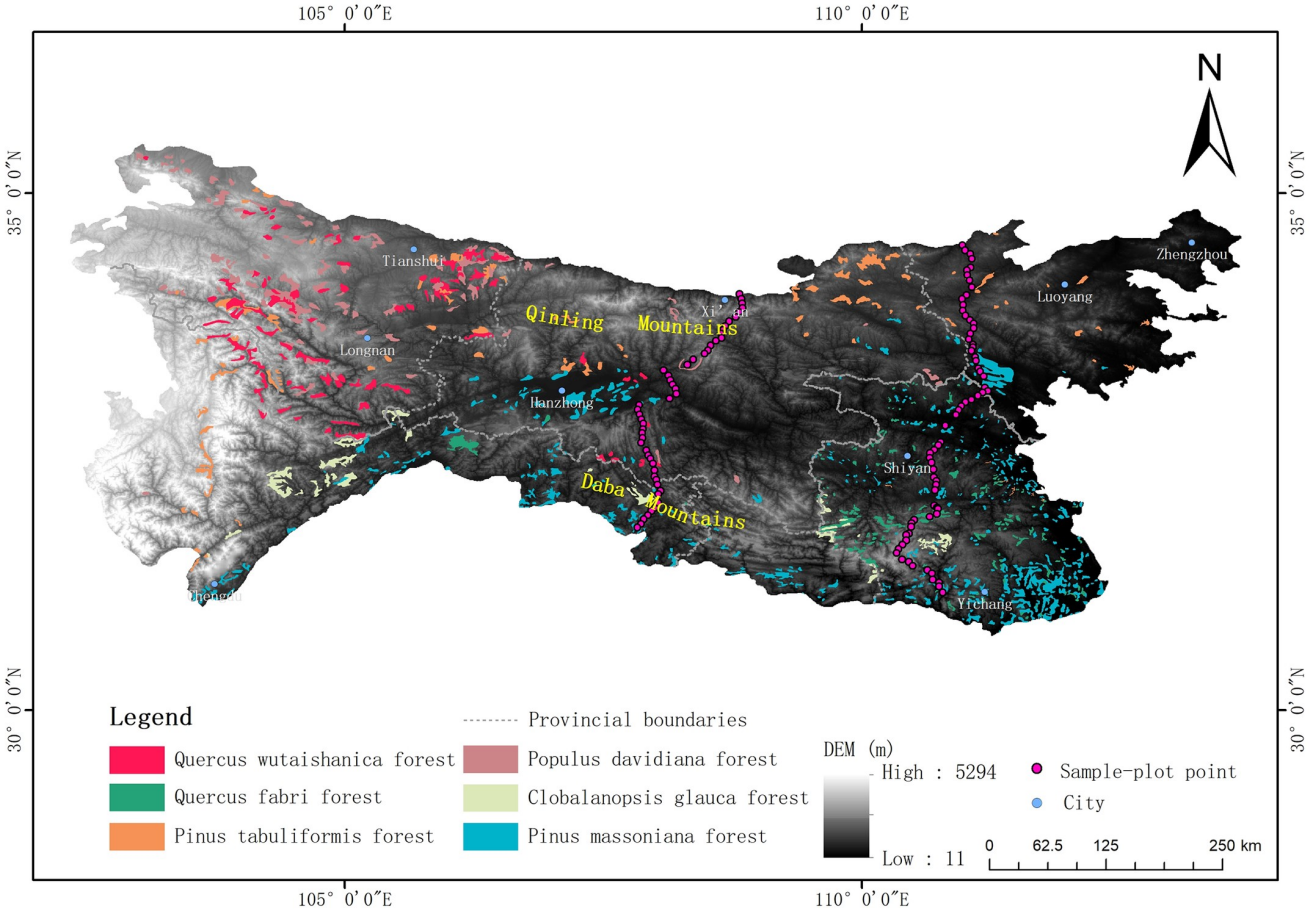
Spatial distribution data of vegetation and plant community survey data.

The datasets were extracted from the Chinese vegetation map (1:1,000,000)(downloaded from the website of http://www.resdc.cn/) [41], and temperate vegetation (Pinus tabuliformis forest, Quercus wutaishanica forest, and Populus davidiana forest) and subtropical vegetation (Pinus massoniana forest, Quercus fabri forest, and Cyclobalanopsis glauca forest) were selected as the vegetation indexes in this study (shown as Fig 2). According to the “Flora Republicae Popularis Sinicae”, these six vegetation types represent the most typical subtropical and temperate vegetation [42]. It was mainly used to analyze the spatial distribution pattern of typical subtropical and temperate vegetation, and delimit boundaries between subtropical and temperate zones. In detail, the six vegetation typess were classified into three groups: Quercus wutaishanica forest and Quercus fabri forest (Group I), Pinus tabuliformis forest and Pinus massoniana forest (Group II), and Populus davidiana forest and Clobalanopsis glauca forest (Group III). Group II was used to delimit the boundary between subtropical coniferous forests and temperate coniferous forests; Group I and Group III were used to delimit the boundaries between subtropical broad-leaved forests and temperate broad-leaved forests.

#### Meteorological data

WorldClim 2.1 climate data were used as meteorological data [43] and included monthly temperature (minimum, maximum and average) and precipitation with a resolution of 1 km² (downloaded from the website of http://www.worldclim.org/). These data were generated using the interpolation method of thin-plate spline based on 9000-60000 meteorological stations and its covariates, including elevation, distance to the coast and various covariates obtained with the MODIS satellite platform. The temporal range for station data was between 1970 and 2000. All data were aggregated to monthly climate averages. A total of 12 images are available for each variable.

#### Meteorological background data of China

The data included the annual average temperature, the annual average precipitation, the accumulated temperature with ≥0°C and the accumulated temperature with ≥10°C with a resolution of 500 m² (downloaded from the website of http://www.resdc.cn/). The data were generated by the interpolation method of inverse distance weighted averages based on data from 1915 national meteorological stations. The meteorological background data were then resampled to 1-km² resolution by calculating the average value with each 1-km pixel. All data were aggregated into one data product. A total of 1 image is available for each variable.

#### Elevation model (DEM)

SRTM (Shuttle Radar Topography Mission) data were used as elevation data. SRTM data are jointly measured by NASA and the National Bureau of Surveying and Mapping (NIMA) with a resolution of 90 m². These data are obtained from the Geospatial Data Cloud (GDC) via downloading from http://www.gscloud.cn/. The DEM was then resampled to 1-km² resolution by calculating the average elevation within each 1-km pixel.

#### Soil data

The spatial distribution data of soil texture in China were collected from the Resource and Environment Data Cloud Platform (http://www.resdc.cn/). The data included the spatial distribution of organic content, nitrogen content, phosphorus content, sand content, silt content, clay content, and soil type with a resolution of 1 km². The data were generated by scanning and digitizing based on “The Second National Soil Survey” and “1:1,000,000 Soil Type Map in the People’s Republic of China”.

#### Plant community survey data

The plant community survey data consisting of 121 quadrats were independently collected in QDM by the “China’s North-South Transitional Zone Comprehensive Scientific Investigation” project (20 m×20 m quadrat area). In total, 71 quadrats were located east of the study area, and 50 quadrats were located in the middle. The attributes of each quadrat included vegetation types, the number of each vegetation type, height, and breast diameter size. Analysis of the real distribution of representative vegetation from the quadrats data can provide effective validation support for our research results.

### Method

The spatial distribution pattern of zonal vegetation is a natural indicator of long-term environmental characteristics [29, 30]. Therefore, the spatial distribution patterns of typical subtropical vegetation and typical temperate vegetation are consistent with the spatial distribution of subtropical and temperate climates. To extract the boundary between the subtropical and temperate zone, in this paper, the suitable distribution of different vegetation types was calculated based on the Geodetector-SVM model. The spatial distribution of various vegetation types was comprehensively analyzed to delimit the subtropical northern boundary and identify the location of the transition zone.

**Fig 3:**
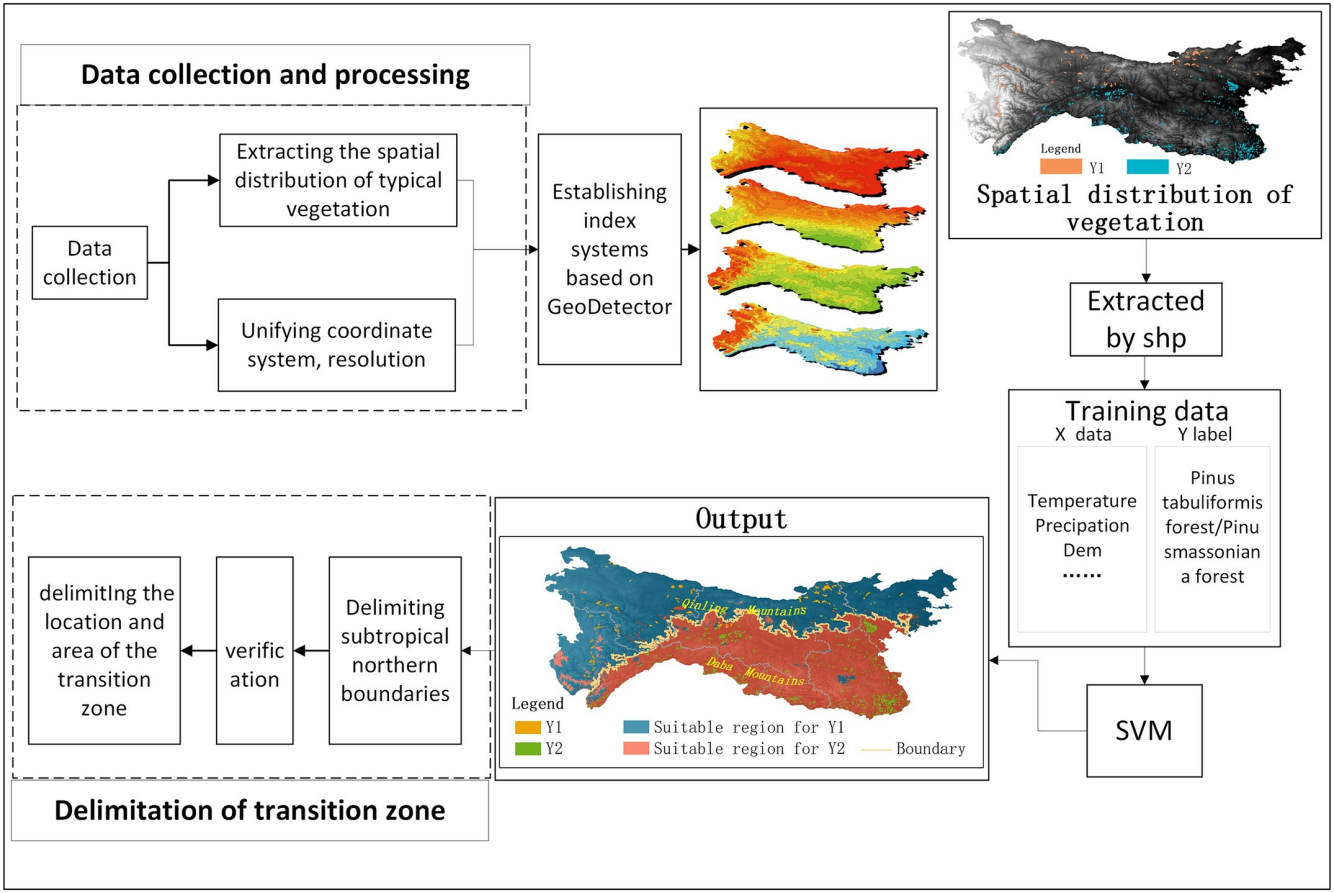
Workflow of the identification of transition zone.

The identifying process of the transition zone was divided into four steps (As shown in Fig 3). The first step was data preprocessing. The spatial distribution of six representative vegetation types were extracted from the Chinese vegetation map (1:1,000,000). The coordinate system of covariates (climatic factors, dem and soil data) was unified to WGS-1984, and the data were resampled to a 1-km² resolution by calculating the average elevation within each 1-km pixel. The second step involved establishing multiple index systems for different vegetation types. The main regionalization indexes of previous regionalization schemes were as follows: the accumulated temperature with ≥0 °C (aat0dem), the accumulated temperature with ≥10 °C (aat10dem), the annual average precipitation (pa), the annual average temperature (tadem), the coldest month average air temperature (meantem01), and the warmest month average air temperature (meantem07) [7–10, 17, 18, 44]. However, in this study, considering the complex topography of QDM and the sensitivity of vegetation to soil, topographic and soil factors were also added into the index system, including DEM, soil organic content, soil nitrogen content, soil phosphorus content, sand content, silt content, clay content, and soil type. Then, to remove redundant information and make full use of potential information, the optimal index system was established using the Geodetector model. The model evaluated the influence of every single factor using q-statistics, which can measure the implication of a single factor on the spatial distribution of the paired vegetation (the paired vegetation refers to the typical subtropical and temperate vegetations, such as Quercus wutaishanica forest and Quercus fabri forest). The higher the value of q-statistics, the stronger impact of a single factor on the spatial distribution of vegetation. According to the previous regionalization scheme, the average q-statistic value is approximately 0.33 between climatic zones [36]. In this study, the variables with q-statistic value greater than 0.3 were selected. Moreover, only four variables in group II (Pinus tabuliformis forest and Pinus massoniana forest) had q-statistic values greater than 0.3. To assure the equality of data for each training session, the first four variables with q-statistic values greater than 0.3 were selected. The third step was to build a comprehensive physical regionalization model based on the SVM to identify the boundary of the subtropical zone and temperate zone in QDM. Specifically, the training sets (including index system) and the labels of training sets (the vegetation types) were used as input in the SVM model, and the zoning maps were output from the model. The experiment was conducted for every paired vegetation, and the subtropical northern boundaries were extracted from each output of the SVM model. The boundary refers to the line between the two partitions of the output of SVM model. The last step was to validate the results using plant community survey data, comprehensively analyze the results, and delimit the location of the transitional zone.

#### Geodetector

The Geodetector model proposed by Wang et al. is a novel and suitable spatial analysis method to measure the degree of spatial stratified heterogeneity [34, 35], which has been widely applied in many fields in recent years [45–47]. In this paper, it was applied to delimit comprehensive physical regionalization. Factor detector, one module of Geodetector, was used to explore factors that were important for different types of vegetation (with q-statistics greater than 0.3) and test their significance. The q-statistics formula is presented as follows:

$$\begin{array}{c} q=\frac{1}{N\sigma^{2}}\sum_{h=1}^{L}N_{h}\sigma_{h}^{2} \end{array}$$ (1)

The study area was stratified into *L* strata, denoted by *h* = 1, 2, &, *L*. In this paper, *h* = 1 or 2, representing the subtropical zone and temperate zone. *N* and *N*_*h*_ are the number of samples in the total study area and the stratum *h*, respectively, *σ*^2^ and $\sigma_{h}^{2}$ denotes the variation of samples in the entire study area and the stratum *h*. A greater q value (the value of *q* ranges from 0 and 1) represents a stronger impact of a single factor on the spatial distribution of vegetation. The greater the *q* value, the stronger the relationship between the explained variable and the explanatory variables, and the larger the nonlinear association within them. In this paper, the explanatory variable are covariates (climatic factors, DEM and soil data), and the explained variables refer to representative vegetation type. For example, Pinus tabuliformis forest and Pinus massoniana forest, which represent the subtropical zone and the warm temperate zone, respectively.

#### SVM

In this paper, the SVM model was used to delimit the subtropical and temperate zones according to the spatial distribution of typical vegetation. The SVM is a new approach developed in recent years and is a robust supervised learning algorithm [37]. Based on nonlinear transformation, SVM maps the dataset to a higher dimensional space. It constructs a hyperplane between two classes that maximizes the margin between the closest training samples of the classes to realize the classification of a dataset [48–50].

Specifically, the four raster layers of covariates represented by the index system are overlapped into a multichannel dataset. The attributes are extracted from raster through the shapefile of the spatial distribution of representative vegetation. Grids with typical vegetation types were used as training sets. If subtropical vegetation is noted in the grid, the label of the grid is 0; if there is temperate vegetation in the grid, the label of the grid is 1. Then, each object of the shapefile has X and Y attributes. Here, X refers to temperature, precipitation, and DEM, whereas Y refers to the vegetation type. The training sets were used as input in the SVM model, and the result is output from the model. The result can be regarded as a zone map.

#### Validation

Validation of the model included the following two aspects: boundary’s accuracy and the analysis of the transitional characteristics.

To verify the plausibility of the boundary, the plant community survey data and the previous climatic boundaries were used for the validation. First, the plant community survey data were used as a reference for the real spatial distribution of vegetation. Specifically, we compared these data with the actual vegetation distribution to judge the degree of plausibility of the results. In this paper, according to the “Flora Republicae Popularis Sinicae” and the plant community survey data [42], typical subtropical vegetation (Platycarya strobilacea forest and Pinus massoniana forest) and typical temperate vegetation (Pinus tabuliformis forest) were selected in this study for validation. In detail, Platycarya strobilacea and Pinus massoniana forests were used to validate the distribution of subtropical broad-leaved forests and subtropical coniferous forests. The plant community survey data with cell sizes of 20 m × 20 m were divided into three grades, including low (2-3), moderate (4-10), and high (11-98), according to the number of Platycarya strobilacea and Pinus massoniana forests. Similarly, Pinus tabuliformis forests were used to verify the distribution of typical temperate coniferous forests. The survey data also were divided into three grades, low (2-10), moderate (11-20), and high (21-60), according to the number of Pinus tabuliformis forests. Second, the boundaries of previous studies were used as a reference for the real subtropical/temperate zone boundary. Compare the differences between them and our result.

The values of each factors in the subtropical zone, transition zone, and temperate zone are calculated respectively, including the coldest month average air temperature (meantem01), the warmest month average air temperature (meantem07), DEM, the annual average precipitation (pa), the accumulated temperature with ≥0°C (aat0dem), and the accumulated temperature with ≥10°C (aat10dem). To analyze the characteristics of the transition zone, we calculate the q-statistic values for evaluating spatial stratified heterogeneity was calculated based on Geodetector model.

## Results and discussion

### Establishment of the typical vegetation index system

The basis of comprehensive physical regionalization is spatial stratified heterogeneity [36]; a higher q represents a stronger spatial stratification. The q-statistics of covariates for every paired vegetation were calculated, as shown in Table 1. With the exception of the sand content and the silt content with q-statistics of 0.471834 and 0.3547604, respectively, which were identified as the driving factors of Group I vegetation (Quercus wutaishanica forest, Quercus fabri forest), no statistically significant spatial heterogeneity of soil-related explanatory factors is noted in the study area, and the q-statistic is generally less than 0.3. This finding indicates that the spatial distribution of vegetation is weakly affected by soil elements. In contrast, the annual average precipitation and the coldest month average air temperature, contributed a remarkable impact, generating q values from 0.47 to 0.63. This finding indicates that both of these factors are the determining factors and significantly affect the spatial distribution of vegetation. Statistically significant spatial heterogeneity of some factors, including the variables of accumulated temperature with ≥10°C, DEM and warmest month average air temperature, is noted in Groups I and II, with a q-statistic greater than 0.3. However, significant heterogeneity is not noted in Group III. This finding indicates that only some vegetation was significantly affected by these factors, whereas others were not.

**Table 1:** Interpretation degree of each explanatory variable.

Variable	Group *I* Quercus wutaishanica forest, Quercus fabri forest	Group *II* Pinus tabuliformis forest, Pinus massoniana forest	Group *III* Populus davidiana forest, Clobalanopsis glauca forest
aat0	0.39281	0.53895	0.300672
aat10	0.422326	0.496743	0.284727
pa	0.578824	0.471186	0.637127
tadem	0.396613	0.542032	0.314665
DEM	0.45271	0.409616	0.217703
meantem01	0.471868	0.639598	0.490474
meantem07	0.45276	0.507524	0.287343
sand content	0.471834	0.124422	0.119135
silt content	0.347604	0.216484	0.141976
clay content	0.273074	0.178613	0.076621
soil organic content	0.005412	0.021158	0.00498
soil phosphorus	0.040388	0.070519	0.096646
soil nitrogen	0.009881	0.020509	0.008813
soil type	0.026083	0.03357	0.003346

**Fig 4:**
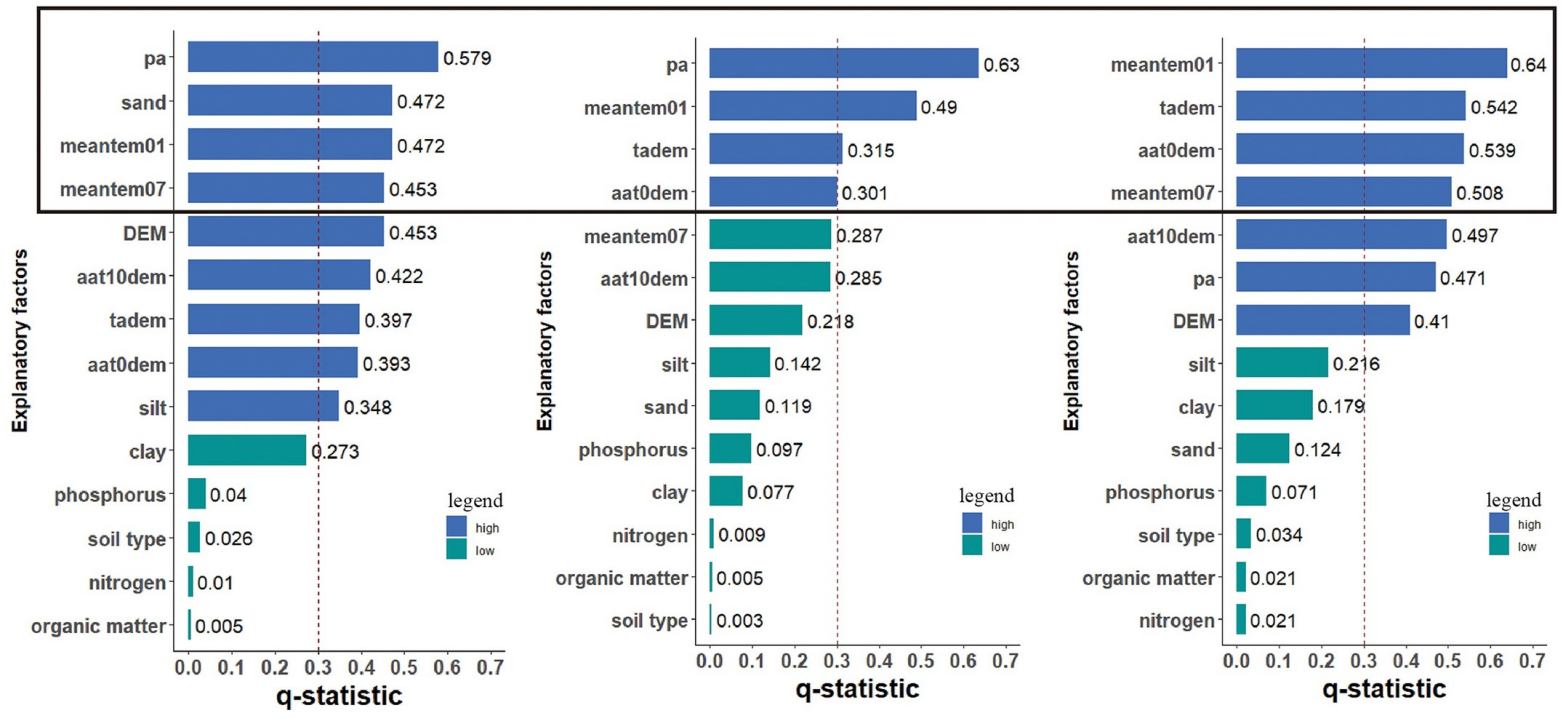
Index system of different vegetation. (a) Index system of Quercus wutaishanica forest and Quercus fabri forest; (b) Index system of Pinus tabuliformis forest and Pinus massoniana forest; (c) Index system of Populus davidiana forest and Clobalanopsis glauca forest.

Based on the sensitivity of different vegetation types to the climatic environment and to remove redundant information, and to assure the equality of data for each training session, the first four variables with q-statistic values greater than 0.3 were selected as the independent variables of the index systems. Thus, three different index systems were generated as shown in Fig 4.

### Extracting boundaries and delimiting the transitional zone

**Fig 5:**
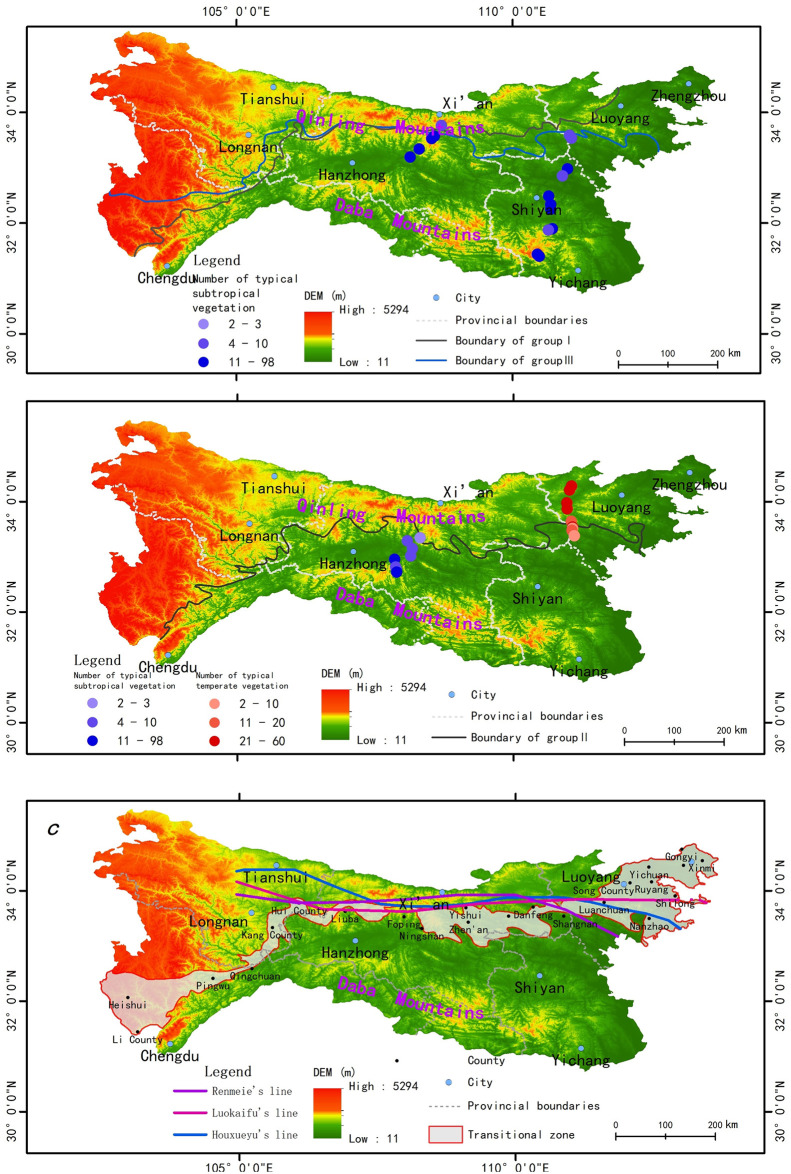
Geodetector-SVM model results and validation. (a) Validation with broad-leaved forests; (b) Validation with coniferous forests; (c) Delimitation of the transition zone.

Three boundaries were output from the model. The three boundaries were not completely consistent, but the trends were similar. Specifically, from west to east, the three boundaries first increase sharply to a high latitude and then tended to be horizontal towards the east. The location of the boundary between the subtropical coniferous forest and the temperate coniferous forest (Fig 5b) was more south than the boundary between the tropical broad-leaved forest and the temperate broad-leaved forest (Fig 5a). The QDM were divided into two major areas, including the northwest, and the southeast, which also refer to the temperate and the subtropical zones, by the boundaries. The similarities and differences among the three boundaries revealed that an exact boundary did not separate the temperate zone and subtropical zone of the QDM; a winding transitional zone was generated. In this paper, the transition zone was delimited by the composition of three boundaries. As shown in Fig 5c, from west to east, the transition zone mainly passes through the cities of Heishui County, Kang County, Liuba County, Shanyang County, and Yichuan County and is, approximately 30 kilometers wide. The characteristics of vegetation gradually changed from south to north. Use of the concept of “the transitional zone” to address the issue of delimiting the temperate zone from the subtropical zone, is more helpful in understanding the synergistic variation of various vegetation, climate, and biological distributions.

### Validation

#### Boundary’s accuracy

The boundaries extracted by this study were divided into two groups: the broad-leaved forest and the coniferous forest (Fig 5a and 5b, respectively). The plant community survey data were used as a reference to represent the real distribution of vegetation types. Compared with these data, the SVM results were highly consistent with the plant community survey data. Specifically, as shown in Fig 5a, the number of typical subtropical broad-leaved vegetation types (Platycarya strobilacea forest) increases gradually from the south to the boundary. At the same time, there is no Platycarya strobilacea forests are not observed north of the boundary. The same pattern is evident in the eastern and central regions of the QDM. This finding confirms that the boundary is consistent with the real distribution of the broad-leaved forest. In addition, as shown in Fig 5b, the number of Pinus tabuliformis forests gradually decrease north of the coniferous boundary, whereas the number of Pinus massoniana forests gradually increase from the coniferous forest boundary to the south. Moreover, the numbers of both forests near the boundary are low. These findings indicate that the delimitation of the coniferous forest boundary is reasonable and consistent with the real distribution characteristics of the coniferous forest. What’s more, the location is approximately the same as the previous climatic boundaries (Renmeie, Luokaifu Houxueyu) (Fig 5c). Is indicate that the delimitation of transition zone is reasonable and consistent with the previous studies.

### Characteristics of the transition zone

As described in the above sections, q-statistics can assess the spatial stratified heterogeneity. Higher q-statistics value indicate increased reliability of the delimitation of the transitional zone. As shown in Fig 5c, the QDM are divided into three regions, namely, the temperate zone, the subtropical zone, and the transition zone. According to the regionalization indexes of all the regionalization schemes, we chose seven of the most commonly used indexes to analyze the characteristics of the transition zone [7–10, 17, 18, 44]. Based on the Geodetector model, a total of 500 random samples were chosen in the three above zones, and the average value and the quantitative q-statistics of the seven indexes were generated (shown in Table 2). On one hand, the values of the seven indexes of the three zones exhibited visible gradient changes. The values of accumulated temperature, annual average temperature, and annual average precipitation gradually increase from the warm temperate zone to the subtropical zone and reach the median value in the transitional zone. The transitional zone acts as a buffer zone; the climate in this region exhibits a distinct transitional character. For example, in the transition zone, the coldest month average temperature is -1.4175°C, the warmest month average temperature is 20.812°C, and the annual precipitation is 803.257 mm.

**Table 2:** Characteristics analysis result of the transition zone.

	meantem07	meantem01	dem	tadem	pa	aat10dem	aat0dem
q	0.35012	0.467286	0.314	0.3778	0.7054	0.351184	0.37433
Temperate zone	16.1323	-5.61467	2323	49.963	6327.66	17805.49	24530.28
Transitional zone	20.812	-1.4175	1422	91.155	8032.57	30051.34	35703.27
Subtropical zone	23.443	1.675667	963	118.12	10035.1	37481.14	43698.89

On the other hand, statistically significant spatial heterogeneity is noted amount the seven indexes, and their q-statistics are generally greater than 0.35. For example, the q-statistic of the average annual precipitation is 0.7054. The average q-statistic of the previous regionalization is approximately 0.33 [36], demonstrating that the transition zone in this study is reasonably credible. These findings indicated that the delimitation results objectively revealed the climate characteristics in the QDM.

### Discussion

#### A new method of the climate regionalization

In this paper, the Geodetector-SVM model was used as a new regionalization method. Compared with commonly used methods of regionalization schemes, this method provides excellent statistical support for the establishment of an index system. Based on q-statistics of the Geodetector model, it is easy to judge which index or variable is more critical for regionalization. In this study, four of 14 waiting indexes were selected as the delimitation indexes of the boundary of subtropical and temperate zones and the transition zone. The results of the boundary and the transition zone were also reasonable. Additionally, due to the differences in the sensitivity of explanatory variables to different vegetation types for a specific boundary, different and appropriate vegetation-environmental indicators can be adopted based on the Geodetector. The Geodetector model can measure the spatial stratified heterogeneity, which satisfies the principle of comprehensive physical regionalization. Three sets of reliable index systems for the boundaries of three couples of vegetation types were acquired based on that model, and redundant information was removed (shown in Fig 3). Moreover, the SVM is a relatively advanced machine learning algorithm, which can efficiently mine potential information from the data. In this study, three reliable boundaries of the vegetation-climatic zone were acquired and verified by the survey quadrat data. These results demonstrate that the Geodetector-SVM model can be used as an efficient method of climate regionalization.

#### The transitional zone, not the boundary

In previous studies, people were focused on delimiting a clear dividing line between the subtropical and warm temperate zones. However, a gradual transition is noted between the subtropical zone and temperate zone. Thus, different boundaries were proposed in the QDM. The sensitivity of diverse vegetation types to climate is different. Thus, the geographical locations of different zonal vegetation types are different. Therefore, focusing on the transitional zone rather than the boundary is more useful to study the direction and intensity of the spatial variation of natural elements in the QDM. As shown in Fig 5a and 5b, the locations of boundaries of subtropical vegetation and temperate vegetation are slightly different, but their trends tend to be similar. These boundaries constitute the transition zone between the subtropical zone and temperate zone. Moreover, the location is approximately the same as the previous climatic boundaries (Renmeie, Luokaifu Houxueyu) (Fig 5c). The transition zone acts as a buffer zone with a distinct transitional character, and this zone is where the temperature, the precipitation, soil type, vegetation change.

#### The limitations of this study

(1) Although vegetation is an indicator of climate conditions, vegetation exhibit a lag with regard to climate change [51]. Additionally, the relationship between boundaries of three vegetation type couples were exclusively considered, and different time scales were not assessed. Based on the result that regionalization might be too rigid, the dynamic change of the climate cannot be reflected via the exclusive use of single-phase data. With global warming, the transition zone between subtropical and temperate regions should change dynamically [52–54]. In future studies, dynamic regionalization models should be established in the study using the time-series datasets. In the context of global changes, the transition zone is sensitive to environmental changes. Therefore, an in-depth study of the composition and distribution characteristics of vegetation and climate within the transition zone can provide strong evidence for regional responses that indicate environmental changes [16]; (2) Given the coarse spatial resolution of the Chinese vegetation map (1:1,000,000) and the difficulties encountered when obtaining vegetation survey data throughout the study region, only three couples of representative vegetation types met the experimental requirements in this study. The delimitation of transition zone should include more types of vegetation, such as shrubs, meadows, and deciduous forests to produce more convincing results; (3) Other explanatory variables affect the spatial distribution of vegetation, such as human activities and extreme weather. Therefore, for more credible regionalization, the selection indicators should consider more comprehensive factors.

## Conclusion

The transitional zone between subtropical and temperate zones was identified by the Geodetector-SVM model based on multiple vegetation-climate indexes, and validation reveals that the transition zone is not only consistent with the distribution of representative vegetation but also similar to the previous climatic boundaries. The Geodetector-SVM model provides good statistical support for the establishment of an index system. The results revealed the following: (1) This paper proposed a workflow for comprehensive physical regionalization and revealed the identification of transition zone rather the division of the boundary can better reflect the transitional character of the QDM and completely reflect the changes in geographical and ecological patterns. (2) From west to east, the transition zone mainly passes through the cities of Heishui County, Kang County, Liuba County, Shanyang County, and Yichuan County, and it is approximately 30 km wide. The average annual precipitation is approximately 803 mm, and the average annual temperature is approximately 9.1°C in the transition zone. The transition zone acts as a buffer zone, and the characteristics of the climatic environment and vegetation types are gradually transformed from a subtropical zone to a temperate zone in this area.

## Supporting information

S1 File Veg data supplemental. The file including the data of Plant community survey and the data of Spatial distribution of vegetation.
(ZIP)
