# Correction: Exploring on the climate regionalization of Qinling-Daba mountains based on Geodetector-SVM model

**DOI:** 10.1371/journal.pone.0246774

**Published:** 2021-02-03

**Authors:** Yufan Hu, Yonghui Yao, Zhixiang Kou

There are errors in the Funding statement. The correct Funding statement is as follows: This work was supported by the National Natural Science Foundation of China (No.41871350) and the Strategic Priority Research Program of Chinese Academy of Sciences (No.XDA20030302).
